# Preparation of 1,4-bis(4-methylstyryl)benzene nanocrystals by a wet process and evaluation of their optical properties

**DOI:** 10.1186/1556-276X-9-16

**Published:** 2014-01-13

**Authors:** Koichi Baba, Kohji Nishida

**Affiliations:** 1 Department of Visual Regenerative Medicine, Osaka University Graduate School of Medicine, 2-2 Yamadaoka, Suita, Osaka 565-0871, Japan; 2 Department of Ophthalmology, Osaka University Graduate School of Medicine, 2-2 Yamadaoka, Suita, Osaka 565-0871, Japan

**Keywords:** Organic nanocrystal, 1,4-bis(4-methylstyryl)benzene, Water dispersion, Bottom-up fabrication technique, Wet process, Optical properties

## Abstract

Single-crystal 1,4-bis(4-methylstyryl)benzene is a promising material for optoelectronic device applications. We demonstrate the preparation of 1,4-bis(4-methylstyryl)benzene nanocrystals by a wet process using a bottom-up reprecipitation technique. Scanning electron microscopy revealed the morphology of the nanocrystals to be sphere-like with an average particle size of about 60 nm. An aqueous dispersion of the nanocrystals was monodisperse and stable with a *ζ*-potential of -41 mV. The peak wavelengths of the absorption and emission spectra of the nanocrystal dispersion were blue and red shifted, respectively, compared with those of tetrahydrofuran solution. Powder X-ray diffraction analysis confirmed the crystallinity of the nanocrystals. The presented 1,4-bis(4-methylstyryl)benzene nanocrystals are expected to be a candidate for a new class of optoelectronic material.

## Background

**Figure 1:**
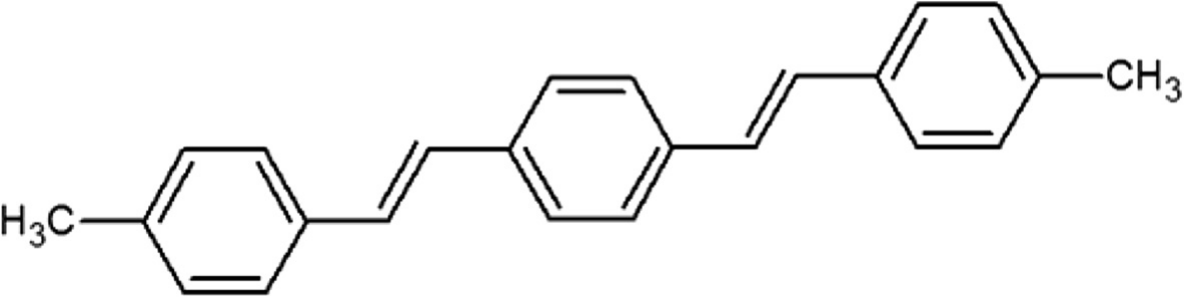
Molecular structure of BSB-Me.

Recently, organic single crystals have attracted considerable attention for optoelectronic device applications because of their high stimulated cross-sections, broad and high-speed nonlinear optical responses, and broad tuning wavelength [1]. In addition, single crystals have the great advantages of high electronic transport and excellent optical properties compared with those of amorphous or polycrystalline thin films [1]. Optoelectronic devices using organic single crystals such as organic field-effect transistors, light-emitting transistors, optically pumped organic semiconductor lasers, and upconversion lasers have therefore been successfully demonstrated [1-5]. Styrylbenzene derivatives are particularly promising candidates for organic transistor and laser oscillation materials. Kabe et al. demonstrated an amplified spontaneous emission from single-crystal 1,4-bis(4-methylstyryl)benzene (BSB-Me) and also studied an organic light-emitting diode using BSB-Me single nanocrystals (the molecular structure of BSB-Me is shown in Figure 1) [6,7]. Yang et al. prepared high-quality, large organic crystals of BSB-Me using an improved physical vapor growth technique and investigated their optical gain properties [1].

In contrast, we have investigated the preparation and evaluated the properties of nano-sized organic crystals, i.e., organic nanocrystals [8-11]. Organic nanocrystals show unique physicochemical properties different from those of the molecular and bulk crystal states [12-15]. Organic nanocrystals have been broadly used as optoelectronic materials as well as biomedical materials [16-22]. Recently, Fang et al. demonstrated the preparation of BSB-Me nanocrystals using a femtosecond laser-induced forward transfer method [23,24]. The BSB-Me nanocrystals were directly deposited on a substrate to form a nanocrystal film, and their size and morphology were investigated as functions of applied laser fluence. The use of BSB-Me nanocrystals will be a promising approach for organic crystal device applications in the near future. However, according to Fang's report, the morphology of the prepared BSB-Me nanocrystals were multifarious, i.e., while most nanoparticles were cubic in geometry, others were tetrahedral shaped, truncated cubes, and truncated tetrahedra [23]. To fabricate high-quality optical devices, such nanocrystals should ideally be homogenous in shape and in size because their optical properties are strongly affected by the crystal morphology. Additionally, there is a serious problem that the yields of nanoparticles prepared by laser ablation are smaller than those obtained by other nanoparticle synthesis methods because the nanocrystals are formed only in the small laser-irradiated spot [25]. This is a weak point when considering mass production for device fabrication. Furthermore, the output power of laser ablation is not suitable for organic compounds because the high energy may degrade them [26,27]. Wet processes using bottom-up techniques overcome these disadvantages. The solvent exchange method, known as the reprecipitation method, is especially suitable for preparing organic nanocrystals [18,28]. Unlike laser ablation, no excess energy is necessary to form the organic nanocrystals, and bulk production is possible [29]. Following a previously reported study, it is possible to prepare a nanocrystal-layered thin film for optical devices using the reprecipitation method [30]. Instead of top-down laser ablation, the alternative approach of this bottom-up wet process is an attractive prospect for preparing BSB-Me nanocrystals.

The aim of this study is to demonstrate the preparation of BSB-Me nanocrystals having narrow size distribution with singular morphology by means of a bottom-up, wet process using the reprecipitation method. This method makes it possible to control the particle size and morphology of the nanocrystals. We prepared BSB-Me nanocrystal dispersions in water, and investigated the size, morphology, optical properties, and powder X-ray diffraction pattern of the nanocrystals.

## Methods

### Materials

BSB-Me (>98.0%) was purchased from Tokyo Chemical Industry Co., Ltd. (Tokyo, Japan) and used without further purification. Tetrahydrofuran (THF) (>99.5%) was purchased from Wako Pure Chemical Industries, Ltd. (Tokyo, Japan). Purified water (18.2 MΩ) was obtained from a Milli-Q A-10 (Millipore, Tokyo, Japan).

### Nanocrystal preparation

BSB-Me was dissolved in THF (2 mM) at 50°C, and 100 μl of the solution was injected into vigorously stirred (1,500 rpm) poor solvent water (10 ml at 24°C) using a microsyringe. As a result, the BSB-Me suddenly precipitated to form dispersed nanocrystals. Syringe filter (pore size 1.2 μm; Minisart®, Sartorius Stedim Biotech, NY, USA) was used to remove small degree of aggregates from the nanocrystal dispersion.

### Evaluation

The particle size and morphology of the BSB-Me nanocrystals were evaluated using scanning electron microscopy (SEM; JSM-6510LA, JEOL, Tokyo, Japan). To prepare specimens for imaging, the nanocrystals were collected from the water dispersion using suction filtration with a membrane filter (0.05-μm pore size), followed by platinum sputter coating (JFC-1600, JEOL). The average particle size, size distribution, and *ζ*-potential of the nanocrystal dispersion were evaluated using an ELSZ-1000 zeta-potential and particle size analyzer (Otsuka Electronics Co., Ltd., Osaka, Japan). Ultraviolet-visible (UV-vis) absorption spectra and fluorescence spectra were measured using a V-550 UV/vis spectrophotometer (JASCO, Tokyo, Japan) and F-2500 fluorescence spectrophotometer (Hitachi, Tokyo, Japan), respectively.

## Results and discussion

**Figure 2:**
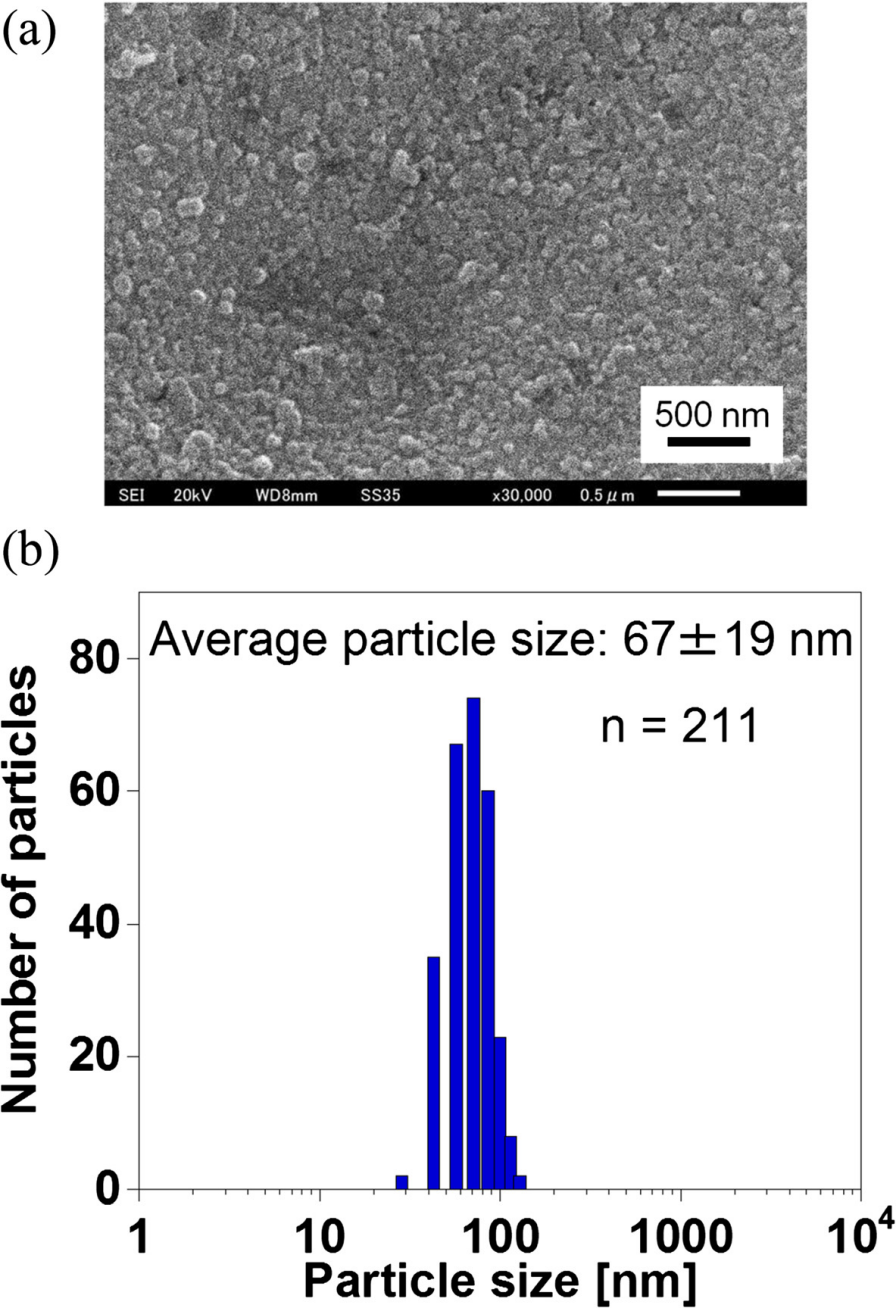
**SEM image of the BSB-Me nanocrystals and their average particle size.** SEM image of BSB-Me nanocrystals **(a)** and average particle size obtained by measuring the size of particles from SEM picture **(b)**. The counted number of particles was *n* = 211. The average particle size was 67 ± 19 nm.

**Figure 3:**
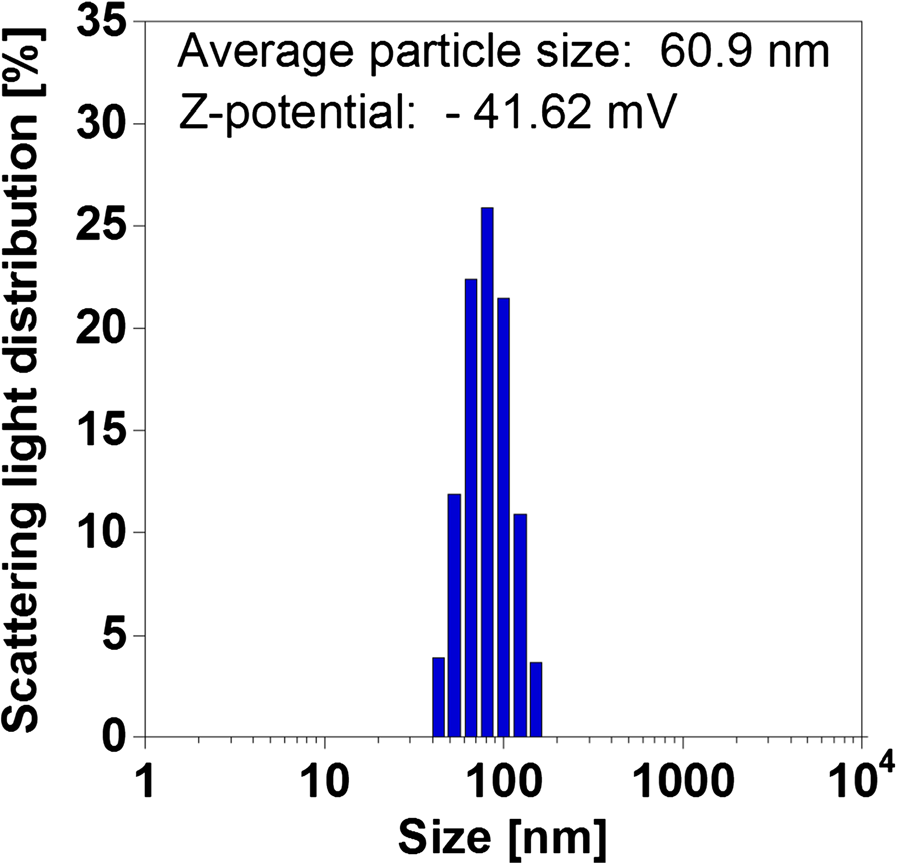
**Average particle size and *ζ*-potential of BSB-Me nanocrystal water dispersion.**

The morphology and particle size of the BSB-Me nanocrystals were investigated using SEM. The nanocrystals were found to be sphere-like and had an apparent average particle size with standard deviation of 67 ± 19 nm. The average particle size was obtained by measuring the particle sizes using the ruler from the SEM picture (the counted particle number was *n* = 211) (Figure 2a,b). The actual particle size, size distribution, and *ζ*-potential of the nanocrystals in the dispersion were investigated using the ELSZ-1000ZS analyzer (Figure 3). The average particle size was 60.9 nm, which was analyzed by cumulant analysis method, in good agreement with that observed by SEM. The *ζ*-potential was -41.62 mV, negative enough to make a stable dispersion. Thus, we succeeded in preparing the BSB-Me nanocrystals stable in aqueous dispersion and with homogenous particle size and morphology.

**Figure 4:**
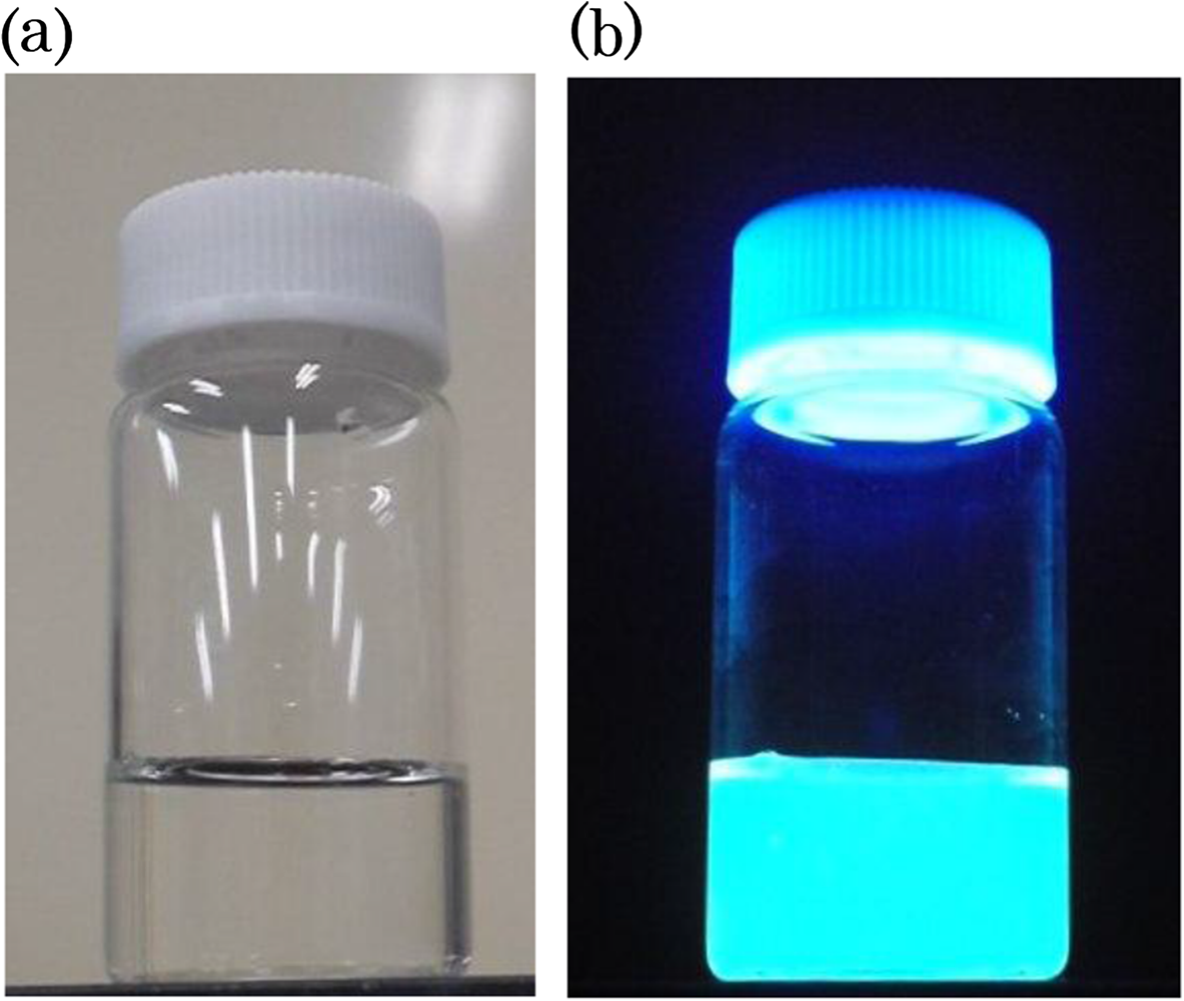
Imaging pictures of BSB-Me nanocrystal water dispersion with (a) and without (b) fluorescence.

**Figure 5:**
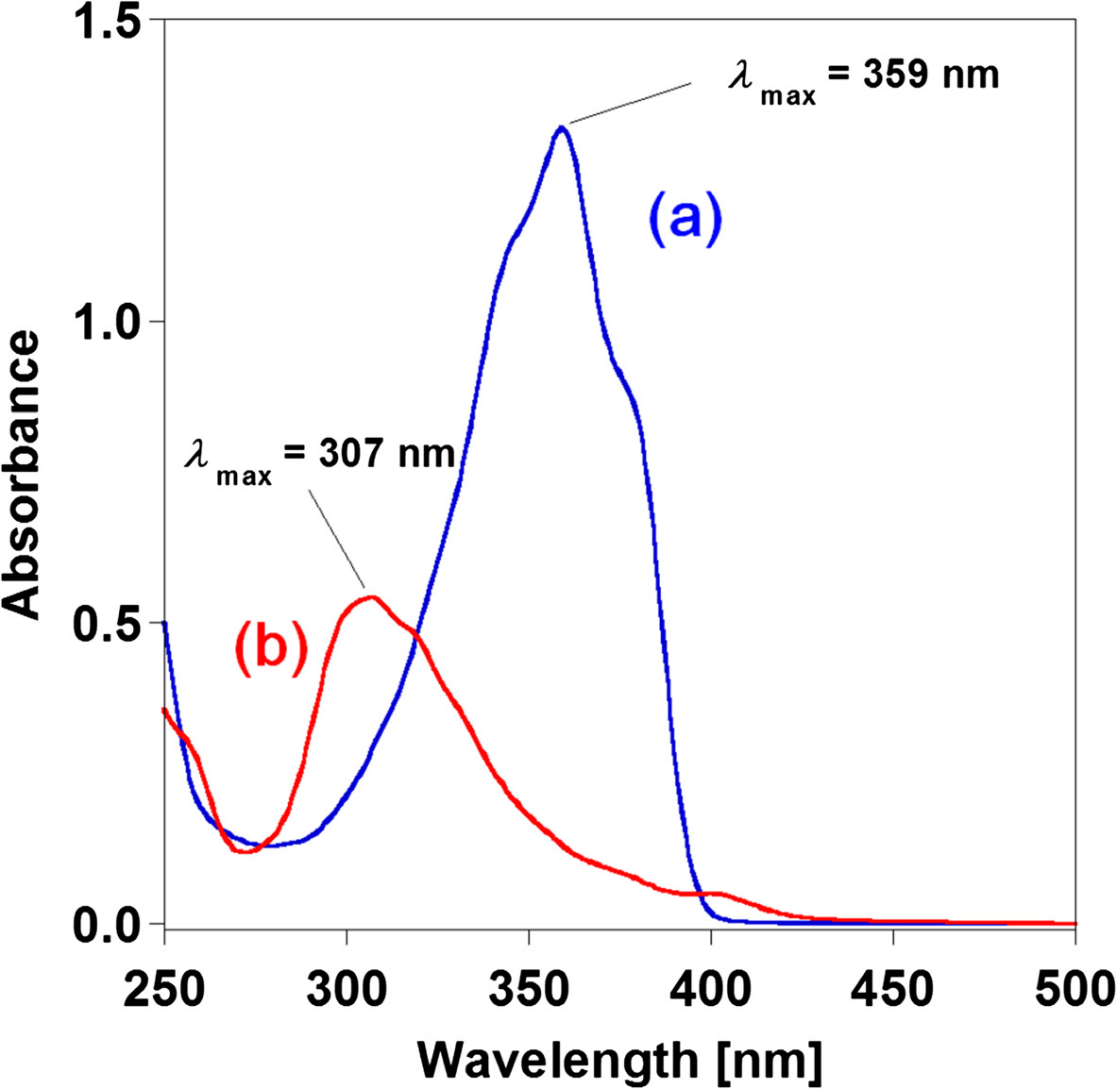
Absorption spectra of BSB-Me THF solution (a) and BSB-Me nanocrystal water dispersion (b).

**Figure 6:**
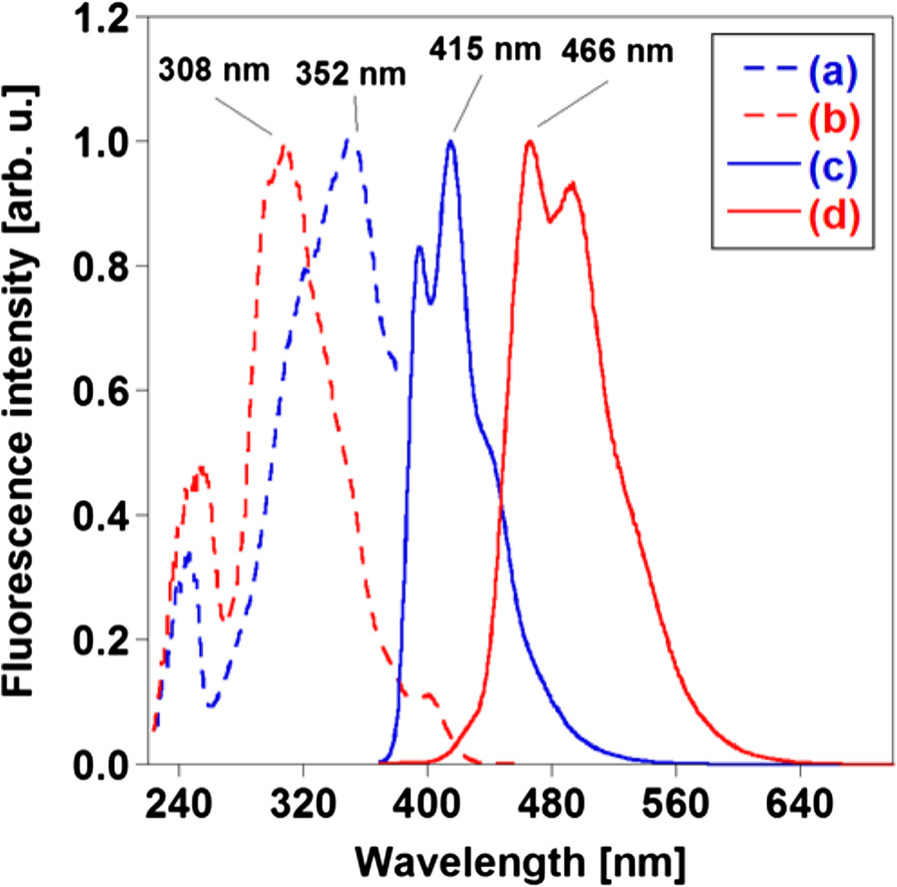
**Fluorescence spectra of BSB-Me THF solution (a, c) and BSB-Me nanocrystal water dispersion (b, d).** Excitation spectra are (a) and (b), which were measured at 395 and 465 nm, respectively. Emission spectra are (c) and (d), which were excited at 350 and 310 nm, respectively.

Photographic images of the BSB-Me nanocrystal dispersion with and without fluorescence are shown in Figure 4. Blue-green fluorescence was observed in the nanocrystal dispersion when it was excited at 365 nm using a UV lamp (SPECTROLINE®, Spectronics Corp., Westbury, NY, USA). Absorption spectra measurements of the BSB-Me THF solution and the aqueous BSB-Me nanocrystal dispersion revealed a blue shift of the maximum absorption peak of the nanocrystal dispersion (*λ*_max_ = 307 nm) compared with that of the THF solution (*λ*_max_ = 359 nm) (Figure 5). Varghese et al. reported that the absorption blue shift in distyrylbenzene single crystals occurs in H-aggregates of herringbone-forming systems, where the long molecular axes are oriented in parallel. However, the short axes are inclined to each other, thus minimizing *π*-*π* overlap. Hence, this side-by-side intralayer orients the transition dipole moments that constitute the main optical absorption band of distyrylbenzene (S0 → S1), leading to a blue shift compared with in solution [31]. The blue shift of the BSB-Me nanocrystal may occur by the same mechanism. Kabe et al. also reported that BSB-Me single crystals have a quasi-planar conformation because of a lack of steric repulsion. This planar structure induces strong supramolecular interactions, which cause the molecules to arrange layer by layer into the well-known herringbone structure [6]. This herringbone forming should affect the emission from the nanocrystals. The emission spectrum of the nanocrystal state showed a red shift (*λ*_max_ = 466 nm) compared with that of the solution state (*λ*_max_ = 415 nm) (Figure 6). This means that the red shifted emission occurred with suppressed high-energy features and a small radiative rate, in other words, indicating the presence of intermolecular interaction in the solid-state aggregated environments, as explained by Varghese et al. [31] and Kabe et al. [6]. The peak wavelength of the excitation spectra of the nanocrystal dispersion (*λ*_max_ = 308 nm) and the THF solution (*λ*_max_ = 359 nm) almost corresponded to those of the respective absorption spectra (Figures 5 and 6).

**Figure 7:**
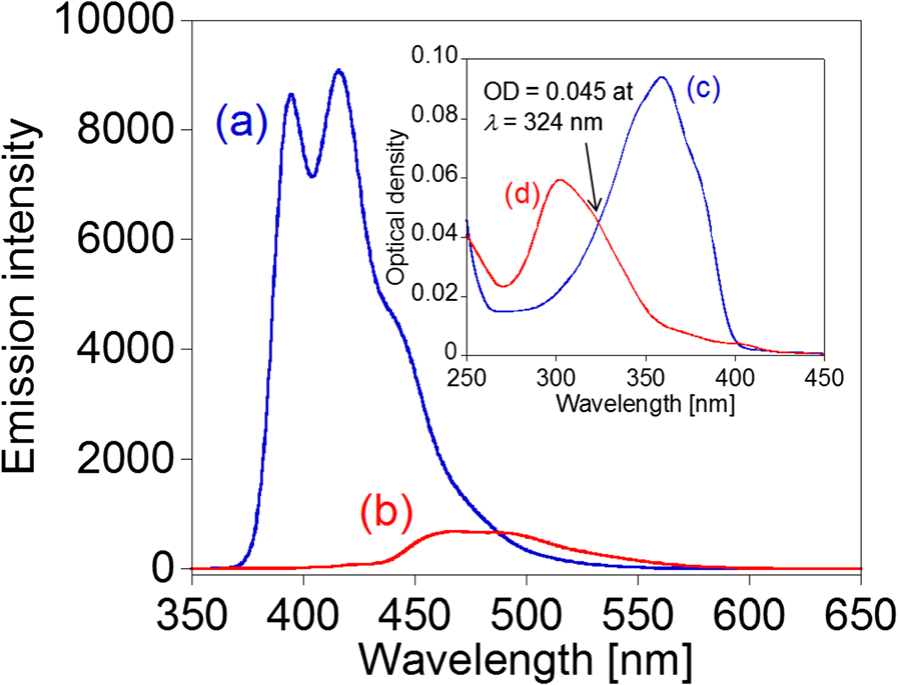
**Emission and absorption spectra of BSB-Me dichloromethane solution and BSB-Me nanocrystal water dispersion.** Emission spectra of BSB-Me dichloromethane solution (a) and BSB-Me nanocrystal water dispersion (b). The excitation wavelength was 324 nm for each spectrum. The integrated intensity (areas) of the spectra was calculated as 528,826 for (a) and 58,884 for (b). Inset: the absorption spectra of the BSB-Me dichloromethane solution (c) and BSB-Me nanocrystal water dispersion (d), where both samples had the same optical density of 0.045 at 324-nm wavelength.

To investigate the photoluminescence efficiency of the BSB-Me nanocrystal water dispersion, we estimated its photoluminescence quantum yield. The manner to estimate the quantum yield of a fluorophore is by comparison with standards of known quantum yield. We used the standard of BSB-Me dichloromethane solution referred in the literature [6], in which the BSB-Me dichloromethane solution had an absolute photoluminescence quantum yield of 95 ± 1%. The quantum yields of the standards are mostly independent of excitation wavelength, so the standards can be used wherever they display useful absorption [32,33]. Determination of the quantum yield is generally accomplished by comparison of the wavelength integrated intensity of the unknown to that of the standard. The optical density is kept below 0.05 to avoid inner filter effects, or the optical densities of the sample and reference (*r*) are matched at the excitation wavelength. The quantum yield of the unknown is calculated using Equation 1:

(1) $$Q=Q_{R}\frac{I}{I_{R}}\frac{{\mathrm{OD}}_{R}}{\mathrm{OD}}\frac{n^{2}}{{n^{2}}_{R}},$$

where *Q* is the quantum yield, *I* is the integrated intensity (areas) of spectra, OD is the optical density, and *n* is the refractive index. The subscripted *R* refers to the reference fluorophore of known quantum yield. The data of *I* and *OD* were obtained from Figure 7. The quantum yield of the BSB-Me nanocrystal water dispersion, which was calculated using Equation 1, was estimated to be 9.2 ± 0.1% (Table 1).

**Table 1:** Quantum yield, integrated intensity, optical density, and refractive index of the BSB-Me

	**Quantum yield (*Q), *%**	**Integrated intensity (*I *)**^**b**^	**Optical density (*OD *) at *λ =*** **324 nm**^**c**^	**Refractive index (*n *) at 20°C**
BSB-Me dissolved in dichloromethane (1 μM)	95 ± 1^a^	528,826	0.045	1.42
BSB-Me nanocrystal water dispersion (2 μM)	9.2 ± 0.1	58,884	0.045	1.33

^a^The data was obtained from Table one of reference [6]. ^b^The data was obtained from Figure 7 (a and b). ^c^The data was obtained from Figure 7 inset (c and d).

**Figure 8:**
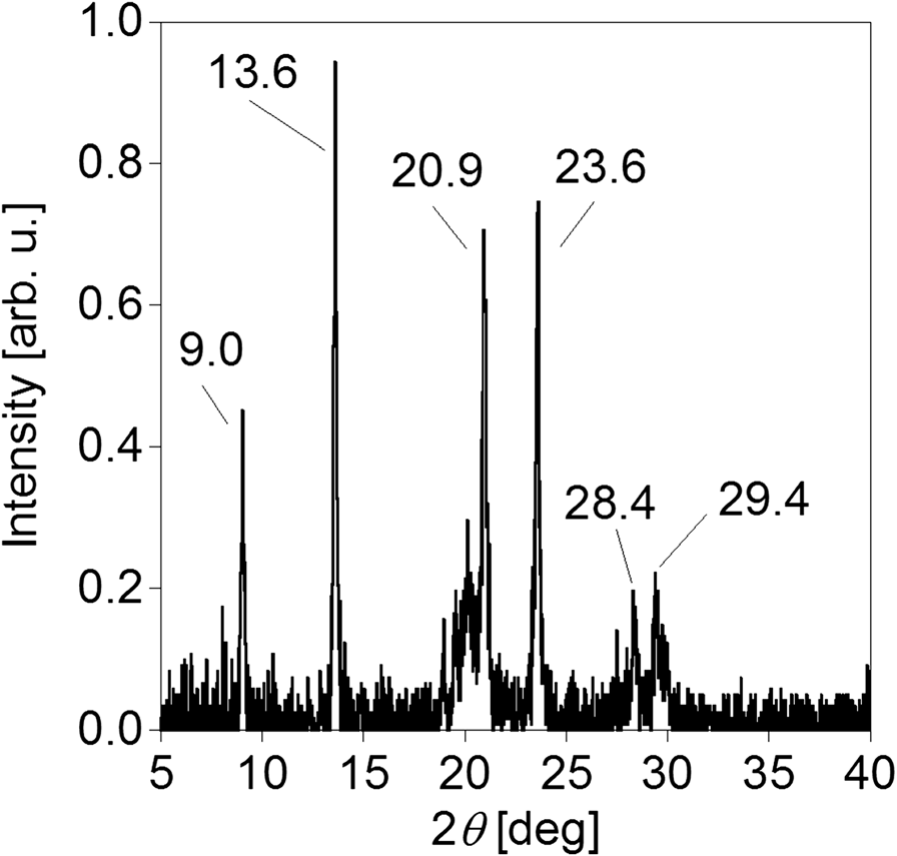
Powder X-ray diffraction analysis of BSB-Me nanocrystals.

The crystallinity of the BSB-Me nanocrystals was confirmed using powder X-ray diffraction analysis (Figure 8). Two strong peaks were observed at 2*θ* = 9.0 and 13.6, corresponding with those previously reported for single bulk crystals [6]. However, interestingly, there were another four strong peaks at 2*θ* = 20.9, 23.6, 28.4, and 29.4 which did not correspond with any previously observed peaks for single crystals [6]. There may be a possibility that a different molecular arrangement to that previously reported for bulk single crystal state was formed in the nanocrystal state. Because the powder X-ray diffraction pattern of the nanocrystals showed (001) refractions as shown in (004) in 2*θ* = 9.0 and (006) in 2*θ* = 13.6, the nanocrystals basically had planar structure, supporting the occurrence of H-aggregation according to the work of Kabe et al. [6]. H-aggregation was also supported by the observed blue shift and red shift in the absorption and emission spectra, respectively, of the nanocrystals. However, because other refractions were observed at 2*θ* = 20.9, 23.6, 28.4, and 29.4, the nanocrystals may have had slightly different crystal structure than the bulk single crystal. Actually, we have previously reported the existence of a softened crystal lattice in nanocrystals [34,35]. A similar softness of the crystal lattice may occur in nanocrystalline BSB-Me. Additionally, in our previous study, there were instances where the crystal structure of the nanocrystal was different from that of bulk crystal [22,36]. That unique optoelectronic properties may occur in nanocrystals compared with bulk single crystals caused by differences in crystal structure is quite interesting, but further investigation is necessary in future work.

## Conclusions

We demonstrated the preparation of a BSB-Me nanocrystal dispersion in water by the reprecipitation method, which is a bottom-up, wet process for preparing organic nanocrystals. SEM observations revealed that the nanocrystals had a sphere-like morphology. The average particle size was 60.9 nm, measured using an ELSZ-1000 zeta-potential and particle size analyzer. The nanocrystal dispersion was stable with a measured *ζ*-potential of -41.62 mV using ELSZ-1000. The blue shift and red shift of maximum peak wavelength were observed in absorption and emission spectra, respectively. This optical feature may have arisen from supramolecular interactions like those caused by the herringbone structure, i.e., H-aggregation, in the nanocrystals. The photoluminescence quantum yield of the BSB-Me nanocrystal water dispersion was estimated to be 9.2 ± 0.1%. Powder X-ray diffraction analysis confirmed the crystallinity of the BSB-Me nanocrystals. In future work, these BSB-Me nanocrystals will be applied to crystalline-based optoelectronic devices. Measuring amplified spontaneous emission and nonlinear optical properties of single nanocrystals will be a particularly interesting topic for the near future. We will also investigate and discuss elsewhere the nanocrystal size distribution using Scherrer's equation based on the data of XRD measurements. Further detailed optical properties such as an absolute photoluminescence quantum yield, fluorescence lifetime, and radiative decay rates of BSB-Me nanocrystals will be discussed elsewhere. Furthermore, fluorescent BSB-Me nanocrystals could be used in biological applications such as fluorescent bioimaging of cells and tissue similar to that in our previous work.

## Competing interests

The authors declare that they have no competing interests.

## Authors’ contributions

KB contributed to the conception of the study, carried out all the experiments, and drafted the manuscript. KN contributed to the interpretation of the data and revision of the manuscript. Both authors read and approved the final manuscript.

## Authors’ information

KB is an Endowed Chair Associate Professor at the Department of Visual Regenerative Medicine, Osaka University Graduate School of Medicine, Japan, and KN is a Professor and a medical doctor at the Department of Ophthalmology, Osaka University Graduate School of Medicine, Japan.
